# Signet-Ring Cell Carcinoma Arising in the Gastric Stump After Duodenopancreatectomy for Ductal Adenocarcinoma of the Pancreas: A Case Report

**DOI:** 10.4137/cmo.s384

**Published:** 2008-03-27

**Authors:** Woubet T Kassahun, Peter Lamesch, Christian Wittekind, Matthias Neid, Jens P Schneider, Joachim Mössner, Johann Hauss

**Affiliations:** 1 Department of Surgery II, University of Leipzig, Liebig Strasse 20, 04103 Leipzig, Germany; 2 Institute of Pathology, University of Leipzig, Liebig Strasse 26, 04103 Leipzig, Germany; 3 Department of Diagnostic Radiology, University of Leipzig, Liebig Strasse 20, 04103 Leipzig, Germany; 4 Department of Internal Medicine II, University of Leipzig, Philipp-Rosenthal Strasse 20, 04103 Leipzig, Germany

**Keywords:** gastric stump carcinoma, peptic ulcer disease, pancreatic carcinoma

## Abstract

The development of malignancy in the gastric stump following surgery for peptic ulcer disease is well recognized. There are also few reports on carcinomas occurring after surgery for malignant gastric disease. However, carcinoma of the gastric stump after duodenopancreatectomy is extremely rare. We describe what we believe to be an unusual case of signet-ring cell carcinoma of the gastric stump developing at the anastomotic site 5 years after duodenopancreatectomy for ductal adenocarcinoma of the pancreatic head. We performed remnant gastrectomy and Roux-en-Y gastrojejunostomy as a curative resection. This experience clearly underlies that g astric stump carcinoma (GSC) may mimic metastatic disease recurrence leading to diagnostic confusion after surgery for malignancy. Although an increased risk of gastric stump carcinoma after pancreatoduodenectomy for pancreatic cancer has not been established, the possibility of such a complication should be kept in mind when evaluating patients after gastric resection who present with symptoms of metastatic disease recurrence years after the primary operation. Investigations should be independent of the entity of the primary disease or its localization, since GSC may well be amenable to surgical cure as demonstrated in the presented case. Outpatient follow up results of the last four years indicated no recurrence in this case.

## Introduction

Cancer risk in the gastric stump after gastric resection for peptic ulcer disease has been reviewed in a number of studies, with reports of increased risk, unchanged risk, or even decreased risk (Ovaska, 1986). However, only 1 case of malignancy (a collision tumor composed of adenocarcinoma and malignant lymphoma) has been reported after duodenopancreatectomy for bile duct cancer (Manabe, 2001). We provide report of a case on a 67 year old patient who developed signet-ring cell carcinoma of the gastric stump at the anastomotic site 5 years after duodenopancreatectomy (Whipple’s procedure) for ductal adenocarcinoma of the pancreatic head. The pathological characteristics and possible etiological aspects are briefly reviewed. Because of the short latency period between the initial gastric surgery and the development of cancer, and the fact that initial surgery was due to pancreatic cancer, rather than benign gastric or duodenal ulcer, this case is unusual and may contribute to expand the cumulative information in the literature.

## Case Report

**Figure 1:**
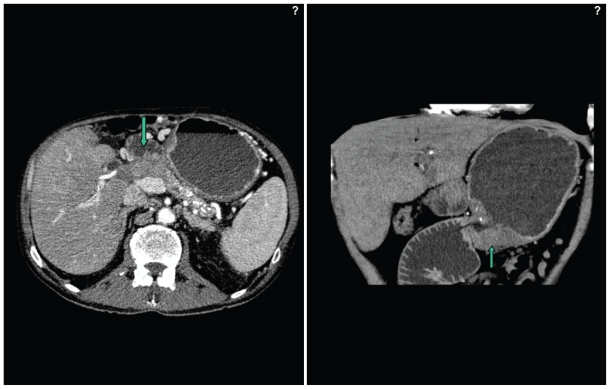
Abdominal CT demonstrating the large, distended fluid-filled gastric remnant, obstructed by a mass at the anastomotic site (arrow) consistent with malignant neoplasm. Disease recurrence of ductal adenocarcinoma of the pancreas was suspected.

**Figure 2:**
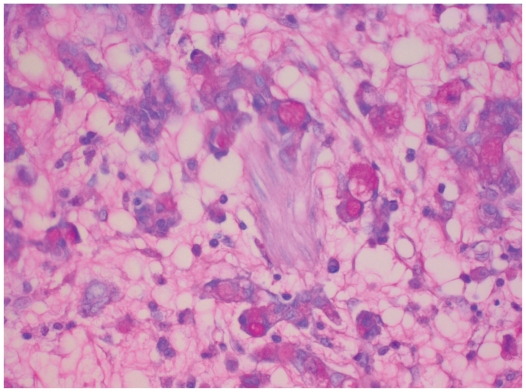
Signet-ring carcinoma cells with high degree of pleomorphism (vary in size and shape). Intracellular mucin displaces the nuclei to the periphery of the tumor cells. Mucicarmine stain, original magnification × 400.

A 67-year old patient was admitted to our department of surgery in October 2003 for evaluation of weight loss. At the age of 62 years, the patient underwent an exploratory laparotomy for a pancreatic head mass with obstructive jaundice that revealed a pancreatic ductal adenocarcinoma. At that time, preoperative contrast-enhanced CT showed a low-attenuation mass in the pancreatic head with signs of chronic pancreatitis of the entire organ. Endoscopic retrograde cholangio-pancreatography (ERCP) showed a malignant obstruction of the intrapancreatic portion of the common bile duct, also known as double-duct sign (proximal obstruction of the common bile and pancreatic ducts). Endoscopy of the upper gastrointestinal tract revealed no suspected lesion in the entire stomach; neither elevated nor depressed mucosal changes were seen. A duo-denopancreatectomy (Whipple’s procedure) was performed. Histology of the specimen confirmed a moderately differentiated pancreatic ductal adenocarcinoma (T3N1Mo, International Union Against Cancer [UICC], stage IIB). One of seventeen peripancreatic lymph nodes was positive for metastatic adenocarcinoma. After uneventful postoperative course, the patient was discharged and periodic follow-up was provided for 5 consecutive years in out patient department. At age of 67 years, the patient experienced increasing fatigue, weight loss, anemia, and intermittent vomiting that worsened by solid food or liquids, and readmitted for further evaluation. Physical examination revealed a horizontal surgical scar supra umbilical on both sides of the abdomen. A non-tender liver was palpable 2 cm below the right costal margin. The spleen and the kidneys were not enlarged. The examination was otherwise unremarkable. The following abnormal laboratory values were obtained: hemoglobin, 5.3 mmol/L (normal, 7.8–10.8 mmol/L), total protein, 51.1 g/L (normal, 66–87 g/L), serum albumin, 24.5 g/L (normal, 35–52 g/L), c-reactive-protein, 77.5 mg/L (normal, <5 mg/L), serum sodium, 129 mmol/L (normal, 135–145 mmol/L), and serum calcium, 1.76 mmol/L (normal, 2.02–2.60 mmol/L). All other laboratory workup results were within the normal range. Endoscopy disclosed obstruction of the gastrojejunal anastomosis. Histology from biopsy specimens on endoscopy revealed an ulcerating adenocarcinoma. An abdominal CT scan performed with intravenous contrast media revealed a mass in the previous location of the pancreatic head (Fig. 1). No further meatastatic disease was found. On the basis of the findings of the clinical examination, endoscopy and abdominal computed tomography, metastatic disease recurrence infiltrating the gastrojejunal anastomosis was strongly suspected. Exploratory laparotomy revealed however, an irregular-shaped elevated lesion 10 × 5 cm in size at the whole circumference of the gastarojejunal anastomosis, obstructing the outlet completely, without the involvement of the pancreatic remnant. Remnant gastrectomy, and Roux-en-Y reconstruction was carried out. Stage IB (UICC, T2bN0M0) ulcerated signet-ring cell carcinoma of the gastric stump was histologically verified (Fig. 2). No metastasis was recognized in all 19 removed lymph nodes, liver or peritoneum. After uneventful postoperative course, the patient was discharged on postoperative day 13 in periodic outpatient follow-up and had been disease free since then.

## Discussion

Cancer newly developed in the gastric stump after partial gastrectomy is worthy of attention not only because it is a typical model of carcinogenesis and a distinct clinical entity but also from the aspect of cancer diagnosis. Its diagnosis is more difficult than in normal whole stomach. Different diagnostic procedures including double contrast study of the upper gastrointestinal tract and computed tomography are incapable of visualizing small lesions. Although endoscopy remains the diagnostic procedure with the highest sensitivity, the decreased size hinders a full-scale examination by endoscopy. Anywhere from 1% to 9% of patients presenting with gastric malignancy have a history of gastric resection for benign disease (Clark, 1985), but whether this truly represents a risk factor for the development of gastric carcinoma is controversial. Some studies have demonstrated more than threefold increase in the number of gastric stump carcinoma observed in those patients followed up for more than 30 years after initial gastric resection for benign disease (Tersmette, 1990). There are also few reports on carcinomas occurring after surgery for malignancy. This condition is mostly seen in patients following distal gastrectomy for gastric cancer (Takeno, 2006).

In about 36% to 90% of the cases, GSC is localized in the anastomotic site (Pointer, 1989). However, neither the exact mechanism for the development of GSC after gastric resection nor the reasons for its frequent localization at the anastomotic site are clearly known. Decreased gastric secretion, decreased acidity, hypoxia, hemodynamic changes, reflux of bile and pancreatic fluid, and changes in bacterial flora are some of the proposed theories to explain how GSC could possibly arise (Lurusso, 2000). Regarding postoperative interval, the gastric mucosa undergoes marked morphologic changes in most patients after gastric surgery in time-dependant manner making postoperative interval the most important determinant of cancer risk following gastric resection. As time goes on, the pronounced mucosal alterations increase parallel to the increasing cancer risk. After 15 years the risk exceeds that of the general population (Safatle-Ribeiro, 1998). As for the reconstruction procedure, it is said that patients undergoing Billroth II-reconstruction develop significantly more carcinomas in the gastric stump than those undergoing Billroth-I reconstruction (Kosaka, 1990). The patient in the presented case was found to have a poorly differentiated signet-ring cell carcinoma in the remnant stomach 5 years after Whipple’s procedure for moderately differentiated ductal adenocarcinoma of the pancreatic head. The paucity of reported cases of malignancy in the remnant stomach after Whipple’s procedure for pancreatic cancer suggests that there may be no increased risk of malignant transformation. This case may, therefore, represent a coincidental finding. Alternatively, GSC in the remnant stomach after duodenpacreatectomy for pancreatic adenocarcinoma may be a late event. In the presented case, reconstruction was performed using Whipple’s procedure, being a Billroth-II type method. Thus the carcinogenesis might have been mainly related to pancreatobiliary reflux in the gastric mucosa. The increased enterogastric reflux and concurrent bacterial overgrowth probably provide the basis for increased mucosal injury creating an environment favorable for the promotion of neoplastic lesions at the anastomotic site. Likewise, distinctive features such as the junction of two different epithelial types at the suture line, and intestinal metaplasia may also have played a role. However, this remains a speculation until causality is undoubtedly proven.

In summary, this experience clearly underlies that GSC may mimic metastatic disease recurrence leading to diagnostic confusion after surgery for malignancy of the pancreatic head. As gastric resection has been found to represent a risk factor for GSC, the possibility of such a complication should be kept in mind when evaluating patients after gastric resection for malignancy of any kind who present with symptoms of metastatic disease recurrence years after the primary operation. Investigations should be independent of the entity of the primary disease or its localization. We would like to emphasize the importance of a thorough diagnostic workup in avoiding pessimistic attitude and confusion of recurrence of the primary disease with GSC, since the later may well be amenable to surgical cure as it was demonstrated in the presented case.
